# Genome-scale comparative analysis for host resistance against sea lice between Atlantic salmon and rainbow trout

**DOI:** 10.1038/s41598-021-92425-3

**Published:** 2021-06-24

**Authors:** Pablo Cáceres, Agustín Barría, Kris A. Christensen, Liane N. Bassini, Katharina Correa, Baltasar Garcia, Jean P. Lhorente, José M. Yáñez

**Affiliations:** 1 Facultad de Ciencias Veterinarias y Pecuarias, Universidad de Chile, Av. Santa Rosa 11735, La Pintana, 8820808 Santiago, Chile; 2 The Roslin Institute and Royal (Dick) School of Veterinary Studies, University of Edinburgh Easter Bush, Midlothian, EH25 9RG UK; 3 Fisheries and Oceans Canada, 4160 Marine Drive, West Vancouver, BC Canada; 4 Escuela de Medicina Veterinaria, Facultad de Ciencias de la Vida, Universidad Andres Bello, Santiago, Chile; 5 School of Agricultural and Veterinary Sciences, UNESP-Sao Paulo State University, Jaboticabal, 14884900 Brazil; 6 Aquainnovo, Cardonal S/N, Puerto Montt, Chile; 7 Núcleo Milenio INVASAL, Concepción, Chile; 8 Center for Research and Innovation in Aquaculture (CRIA), Universidad de Chile, Santiago, Santiago Chile

**Keywords:** Animal breeding, Genome-wide association studies

## Abstract

Sea lice (*Caligus rogercresseyi*) is an ectoparasite which causes major production losses in the salmon aquaculture industry worldwide. Atlantic salmon (*Salmo salar*) and rainbow trout (*Oncorhynchus mykiss*) are two of the most susceptible salmonid species to sea lice infestation. The objectives of this study were to: (1) identify genomic regions associated with resistance to *Caligus rogercresseyi* in Atlantic salmon and rainbow trout by performing single-step Genome-Wide Association studies (ssGWAS), and (2) identify candidate genes related to trait variation based on exploring orthologous genes within the associated regions across species. A total of 2626 Atlantic salmon and 2643 rainbow trout were challenged and genotyped with 50 K and 57 K SNP panels, respectively. We ran two independent ssGWAS for sea lice resistance on each species and identified 7 and 13 regions explaining more than 1% of the genetic variance for the trait, with the most important regions explaining 3% and 2.7% for Atlantic salmon and rainbow trout, respectively. We identified genes associated with immune response, cytoskeleton function, and cell migration when focusing on important genomic regions for each species. Moreover, we found 15 common orthogroups which were present in more than one associated genomic region, within- or between-species; however, only one orthogroup showed a clear potential biological relevance in the response against sea lice. For instance, *dual-specificity protein phosphatase 10-like* (*dusp10*) and *dual-specificity protein phosphatase 8* (*dusp8*) were found in genomic regions associated with lice density in Atlantic salmon and rainbow trout, respectively. *Dusp10* and *dusp8* are modulators of the MAPK pathway and might be involved in the differences of the inflammation response between lice resistant and susceptible fish from both species. Our results provide further knowledge on candidate genes related to sea lice resistance and may help establish better control for sea lice in fish populations.

## Introduction

Sea lice is currently the most harmful parasite for salmon farming worldwide^1^. Economic losses due to different sea lice species are mainly associated with the reduction of feed conversion rate, growth, indirect mortalities and loss of product value. Furthermore, it has been estimated that the global costs for sea lice control have reached $480 million (USD) annually^2^. The two most important sea lice species which generate a considerable negative impact in salmon farming are *Lepeophtheirus salmonis* and *Caligus rogercresseyi*^3^.

*Caligus rogercresseyi*, first described in 2000 by Boxshall and Bravo^4^, is the main sea lice species affecting salmon aquaculture in Chile^5^. *C. rogercresseyi* primarily affects Atlantic salmon (*Salmo salar*) and rainbow trout (*Oncorhynchus mykiss*), while coho salmon (*Oncorhynchus kisutch*) has an innate lower susceptibility to the parasite^6^. The consequences of the infestation by sea lice include skin lesions, osmotic imbalance and greater susceptibility to bacterial and viral infections through the suppression of the immune response by the damage generated in the skin of the host^7^. The parasite life cycle is comprised of eight stages of development^8^: two states of nauplii, one copepod state, four chalimus states and the adult state. The stages of nauplii (I and II) and copepods (infectious stage) are planktonic. The four stages of chalimus (I–IV) are sessile while the adult is a mobile stage^9^.

Recent studies have estimated low to moderate genetic variation for resistance to sea lice in Atlantic salmon populations, with heritabilities ranging from 0.12 to 0.32 and from 0.13 to 0.33 when resistance was defined as the number of parasites fixed in all the fins^7,9,10^, or as the logarithm of the parasite density^11,12^, respectively. A recent study reported a heritability value of 0.09 for sea lice resistance in a rainbow trout breeding population^13^. These results indicate that it is feasible to improve resistance to sea lice in Atlantic salmon and rainbow trout populations by utilizing selective breeding^9,10,14^.

Comparative genomic approaches^15^ allow the identification of genomic similarities between different species, including conserved genes and motifs, traces of genome duplication and gene functions^16^. Traditionally, these analyses are focused on orthologous genes, which are homologous genes present in different species resulting from direct transmission from a common ancestor^16^. To date, comparative genomic studies between salmonids have mainly focused on finding evolutionary similarities in the genetic basis of body and sex related traits, including growth, development and sexual differentiation^17–19^.

A recent study compared results from genome-wide association studies (GWAS) on three salmonid species and identified functional candidate genes involved in resistance to the infection caused by *Piscirickettsia salmonis*, an intracellular bacterium^20^. To date, no studies have aimed at comparing genomic regions associated with resistance to sea lice in salmonid species.

The main objectives of the present study were to: (1) identify genomic regions associated with resistance to *Caligus rogercresseyi* in Atlantic salmon and rainbow trout through GWAS, and (2) identify functional candidate genes potentially related to trait variation through a comparative genomics approach, based on exploring orthologous genes within the associated regions between species.

## Results and discussion

The comparative genomics analysis presented here allowed us to identify groups of orthologues genes and several candidate genes among adjacent single nucleotide polymorphisms (SNP) that explained more than 1% of the genetic variance for resistance to *C. rogercresseyi*. This is the first study aimed at comparing the genomic basis of sea lice resistance in both Atlantic salmon and rainbow trout.

### Sea lice challenge test

There was no difference between the average number of sea lice found on Atlantic salmon or rainbow trout in the experimental challenge (Table 1). An average of 5.9 ± 6.6 and 6.1 ± 4.2 parasites per fish was estimated for Atlantic salmon and rainbow trout, respectively. In terms of the maximum number of parasites, this value varied from 106 parasites in Atlantic salmon to 28 in rainbow trout and, for both species, some animals did not present any parasites. The average weight at the end of the experimental challenge was 278.1 ± 90.3 g (ranging from 104 to 569 g) and 173.1 ± 31.4 g (ranging from 86 to 265 g), for Atlantic salmon and rainbow trout, respectively. Although the average number of parasites was not significantly different between the two species, the difference in the average final weight at the end of each challenge could explain the difference in the maximum number of parasites found (~ 4 times more parasites in Atlantic salmon). The number of parasites counted in each species after the challenge is below the range determined in previous studies. For instance, Ødegård et al.^11^ obtained an average of 20.96 ± 19.68, while Robledo et al.^21^ reported an average of 38 ± 16 sea lice. The lower number of parasites in comparison to these studies is most likely related to differences in the area of counting (whole body surface versus only fins), parasite species (*Lepeophtheirus salmonis* versus *C.rogercresseyi*) and the time of sampling after infestation (8–15 versus 6 days).

**Table 1:** Summary statistics for body weight (BW) and lice count (LC) in Atlantic salmon and rainbow trout.

Species	Mean BW (g)	BW SD (g)	Min BW(g)	Max BW (g)	LC means	LC SD	Min LC	Max LC
*S. salar*	278.1	90.3	104.0	569.0	5.9	6.61	0	106
*O. mykiss*	173.1	31.4	86.0	265	6.1	4.22	0	28

*SD* standard deviation.

**Figure 1:**
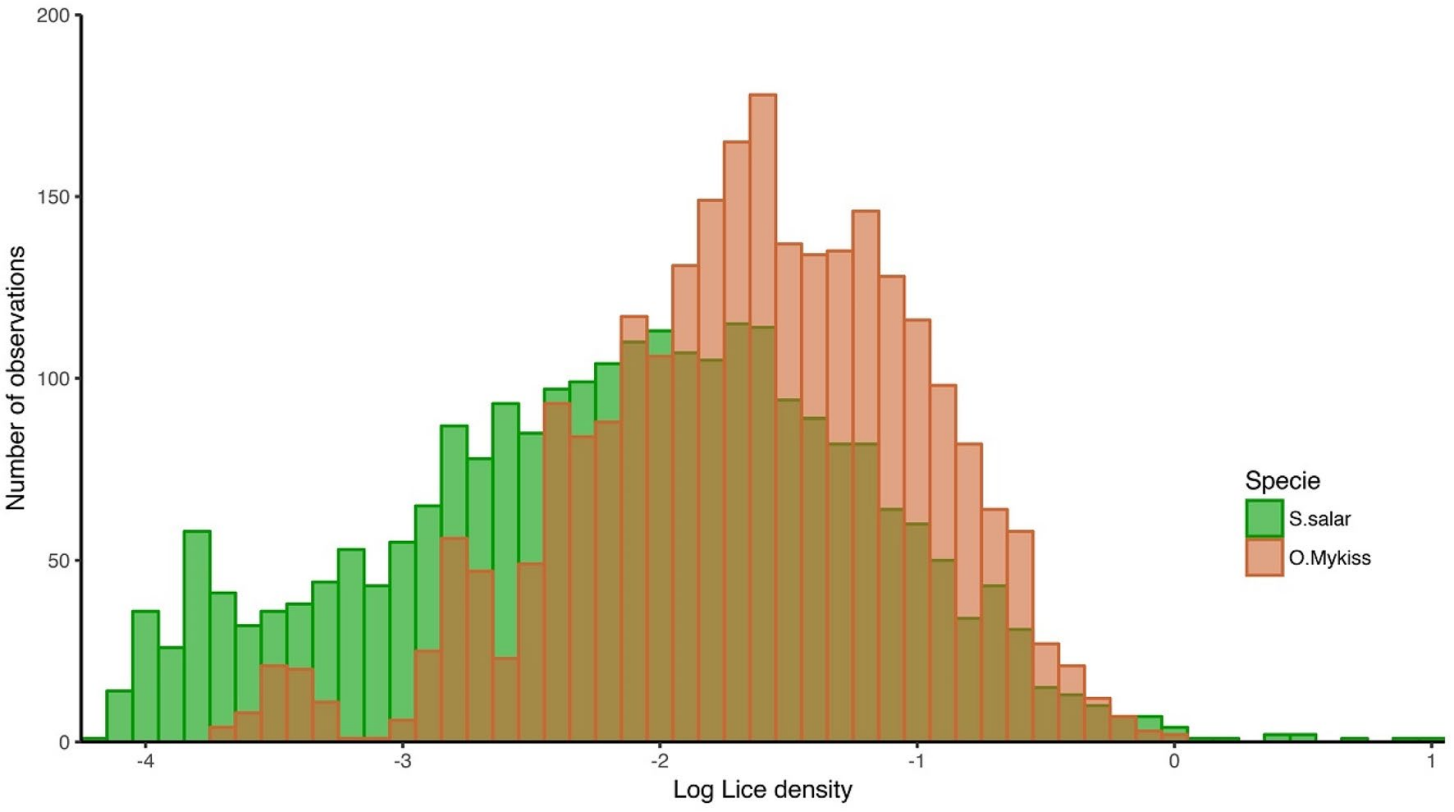
Histogram for log lice density (LogLD) for *S. salar* (green) and *O. mykiss* (orange).

To measure resistance to *C. rogercresseyi*, lice count values were transformed into lice density on the log scale (LogLD), which allows for correction of the number of parasites based on the body weight of each fish^11^. The empirical LogLD distribution for both species is shown in Fig. 1. The range of LogLD for Atlantic salmon was greater than for rainbow trout, varying from − 4.60 to 1.02 and from − 4.06 to 0.02, respectively. The average LogLD was − 2.14 ± 0.9 and − 1.66 ± 0.67 for Atlantic salmon and rainbow trout, respectively, which are similar to those reported in a previous study in a different Atlantic salmon population (between − 1.66 ± 0.73 and − 2.55 ± 0.58)^11^.

### Genotyping and genomic heritabilities

A total of 2040 (77.6%) animals, and 45,117 (96.7%) SNPs passed the genotyping quality control for Atlantic salmon. In the case of rainbow trout, 2466 (93.3%) fish and 27,146 (67.4%) SNPs remained for subsequent analyses. In both species, significant genetic variation for resistance to *C. rogercresseyi* was estimated by using genomic information, with heritability values of 0.19 ± 0.03 and 0.08 ± 0.01 for Atlantic salmon and rainbow trout, respectively (Table 2).

**Table 2:** Summary of heritability (h^2^) for the LogLD trait in both species.

Specie	Additive variance	Residual variance	*h*^2^
*S*. *salar*	0.09 ± 0.01	0.41 ± 0.01	0.19 ± 0.03
*O*. *mykiss*	0.02 ± 0.005	0.27 ± 0.008	0.08 ± 0.01

In previous studies in Atlantic salmon populations, Tsai et al.^12^, estimated genomic heritability values for resistance against *L. salmonis* of 0.22 ± 0.08 and 0.33 ± 0.08, while Ødegård et al.^11^, found heritability values of 0.14 ± 0.03 and 0.13 ± 0.03. Similarly, Yañez et al*.*^10^ and Correa et al*.*^7^ estimated heritability values ranging from 0.10 to 0.12 when defining resistance as the total number of parasites found on all fins using pedigree and genomic information, respectively, and Lhorente et al*.*^9^ estimated heritability values of 0.22 ± 0.06 in Atlantic salmon for total count of sessile sea lice per fish, corrected by body weight in the statistical analysis.

### GWAS

**Figure 2:**
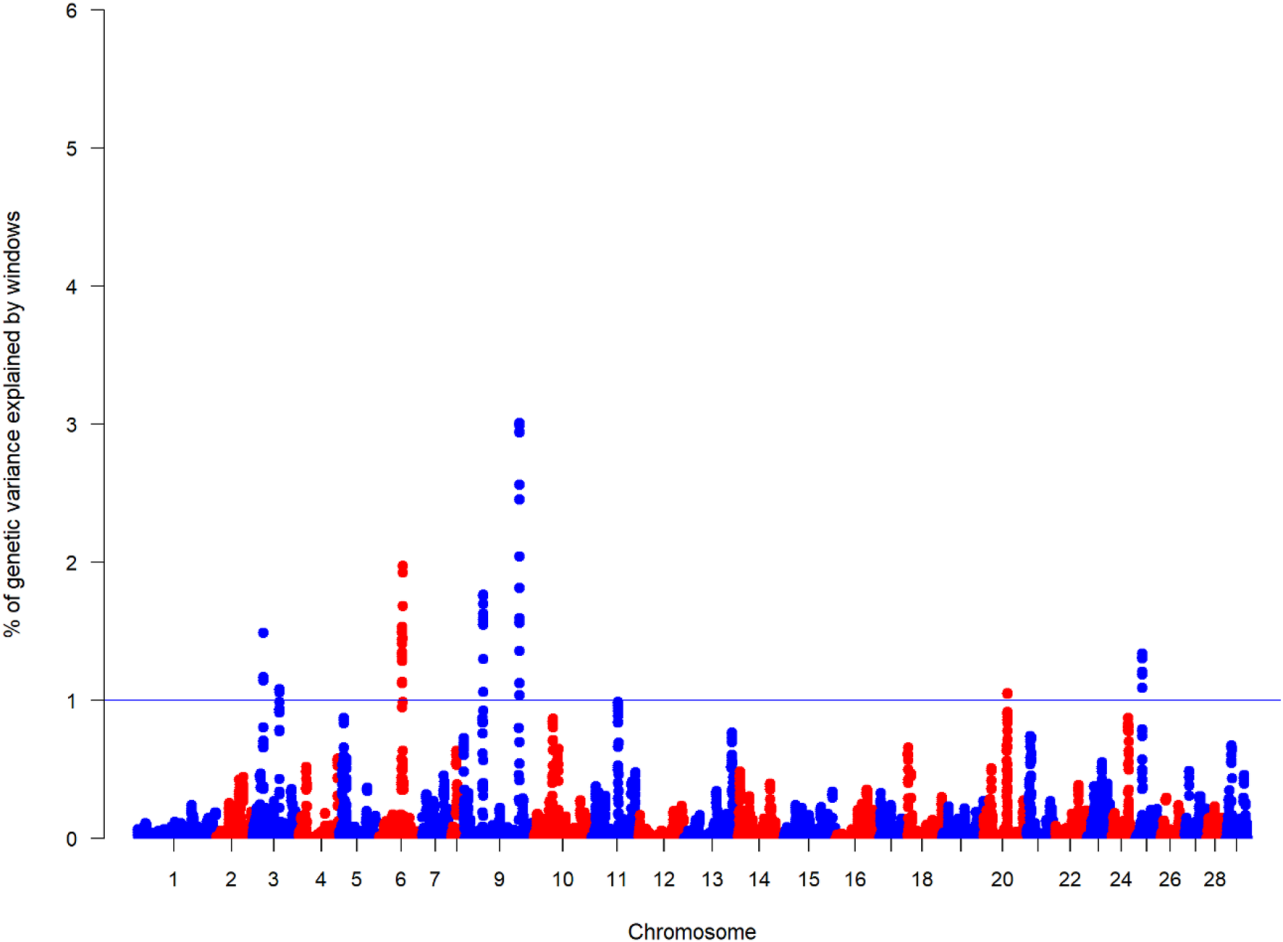
Weighted single-step GWAS (wssGWAS) results for Log lice density (LogLD) in Atlantic salmon. The Manhattan plot represents the genetic variance explained by windows of 20 SNP and the blue horizontal line indicates 1% threshold.

In Atlantic salmon, we found genomic regions explaining more than 1% of the genetic variance for sea lice resistance in five different chromosomes (Fig. 2). Two of these chromosomes (*Ssa3* and *Ssa9*) showed two QTL peaks associated with the trait. Only four SNPs located in *Ssa3*, *Ssa11*, *Ssa14* and *Ssa23* were shown to be significantly associated with the trait at a chromosome-wide level. However, none of these QTLs explained more than 1% of the genetic variance of the trait (Supplementary Figure S1). In general, these regions explained a low percentage of the total genetic variation with a maximum of 3% explained by a single locus. The two SNP windows in *Ssa3* explained 1% and 1.4% of the genetic variance while those found in *Ssa9* explained 1.7% and 3%. Other QTLs found *in Ssa6*, *Ssa20*, and *Ssa25* explained 1.9%, 1.05% and 1.33% of the genetic variance, respectively. Supplementary Table S1 shows the variance explained by each window of SNP in both species.

**Figure 3:**
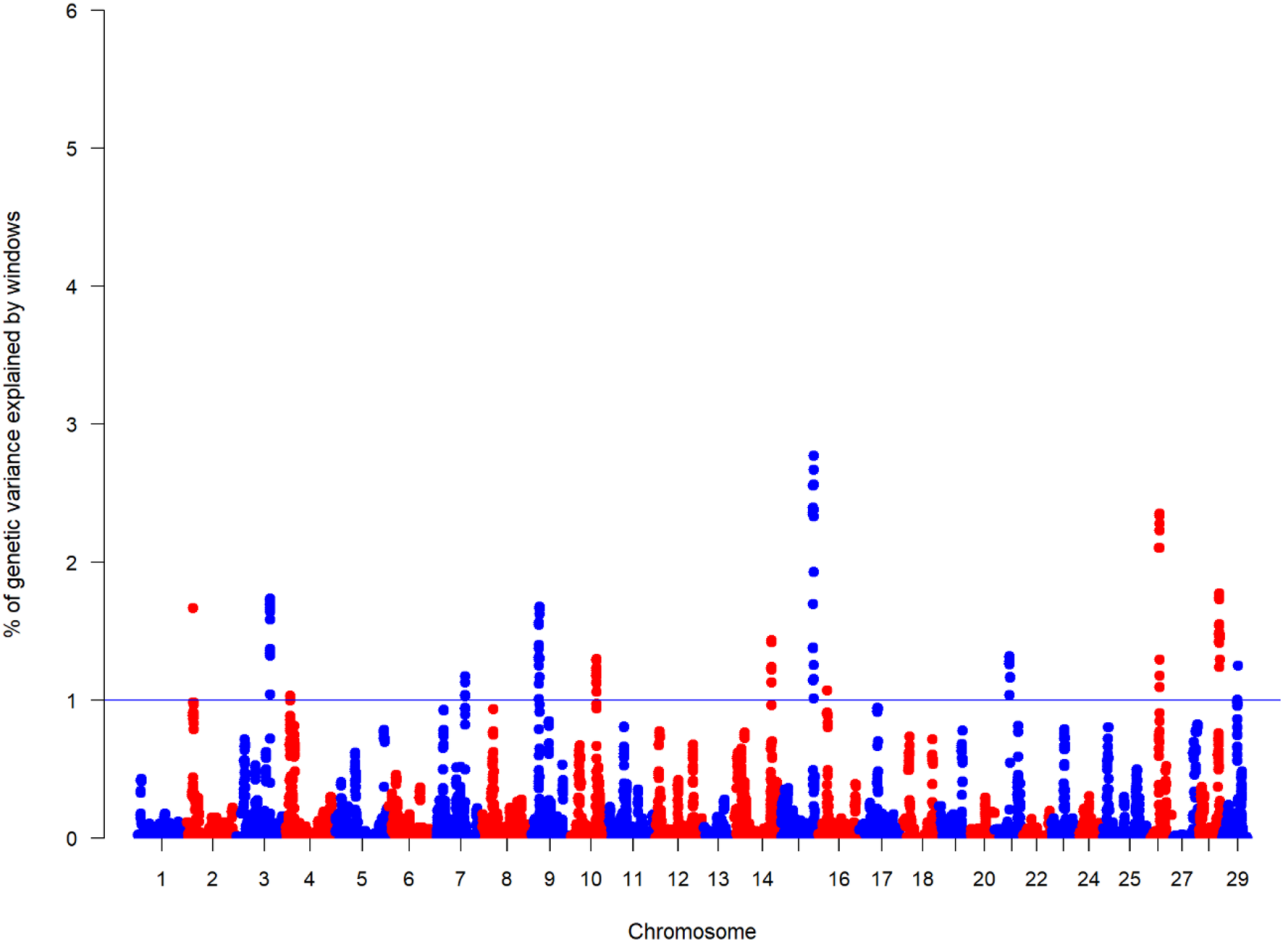
Weighted single-step GWAS (wssGWAS) results for Log lice density (LogLD) in rainbow trout. Manhattan plot represents the genetic variance explained by windows of 20 SNP and the blue horizontal line indicates 1% significance threshold.

In the case of rainbow trout, the wssGWAS for LogLD identified 13 regions located in different chromosomes that exceeded 1% of the total genetic variance for the trait (Fig. 3). Similar to Atlantic salmon, these windows explained a low percentage of the total variance with a minimum of 1% and a maximum of 2.7%, for QTLs located in *Omy17* and *Omy15*, respectively. In addition, three SNPs located in *Omy3*, *Omy6*, *Omy9* showed chromosome-wide significant association with the resistance trait in rainbow trout (Supplementary Figure S2).

Our results suggest that resistance against *C. rogercresseyi* is mainly under polygenic control (i.e., influenced by several genes with small effects) in both species. These results are in agreement with previous studies on sea lice resistance, where a similar genetic architecture was suggested for resistance against *L. salmonis* and *C. royercress*eyi resistance^7,12,22^. Recently Robledo et al.^23^ described and characterized three QTLs related to sea lice resistance in Atlantic salmon by using GWAS and RNA-sequencing approaches. Since sea lice resistance is a polygenic trait, the acceleration of genetic improvement will most likely be best accomplished by employing genomic selection instead of marker-assisted selection or pedigree-based genetic evaluations. For instance, Correa et al.^12^ and Tsai et al.^14^ described an increase of over 22% in the accuracy of estimated breeding values (EBVs) using genomic selection over the use of pedigree-based models in Atlantic salmon^24^.

### Candidate genes

The exploration of the genes within the windows that explained over 1% of the genetic variance for LogLD showed several potential candidate genes that were classified into three groups: related to the immune response, cytoskeleton or metalloproteases. The genes are listed in Tables 3 and 4 for Atlantic salmon and rainbow trout, respectively.

**Table 3:** Candidate genes for sea lice resistance in Atlantic salmon.

Protein name	Gene ID^a^	Location (BP)^b^	Chr^c^	Function
Tenascin R	*tnr*	*22,113,558–22,312,716*	3	Immune response
T-cell activation Rho GTPase-activating protein	*tagap*	47,344,667–47,350,357	6	Immune response
Forkhead box protein N1-like isoform X1	*LOC106612922*	*111,631,268–111,668,978*	9	Immune response
Immunoglobulin superfamily member 11-like	*igsf11*	47,453,435–47,570,948	20	Immune response
Tripartite motif-containing 45	*trim45*	*31,699,662–31,705,320*	25	Immune response
pre-B-cell leukemia transcription factor 1-like	*LOC106607421*	43,977,204–44,037,235	6	Immune response
Bromodomain-containing protein 4-like isoform	*LOC106600922*	*53,482,098–53,526,053*	3	Immune response
PAPPALYSIN-2	*pappa2*	21,972,287–22,050,408	3	Metalloprotease
Carboxypeptidase D	*cdpa*	*111,778,091–111,828,613*	9	Metalloprotease
GEM-interacting protein-like isoform X3	*LOC106600913*	53,672,635–53,709,985	3	Metalloprotease
Epidermal growth factor	*egf*	55,552,926–55,584,698	9	Cytoskeleton
Procollagen galactosyltransferase 1	*LOC106607427*	*43,820,342–43,851,337*	6	Cytoskeleton
Heat shock protein HSP 90-beta	*hs90b*	48,362,362–48,374,359	6	Cytoskeleton
Collagen alpha-1(XXVIII) chain-like	*col28a1*	*13,872,240–13,923,778*	25	Cytoskeleton
Gap junction alpha-4 protein-like	*LOC106611294*	39,373,024–39,373,931	9	Cytoskeleton
Pleckstrin homology domain-containing family H member 1-like	*LOC106611291*	*39,101,628–39,165,084*	9	Cytoskeleton
Serine/threonine-protein kinase OSR1-like	*LOC106600551*	44,726,186–44,781,350	3	Cytoskeleton
Rho-related GTP-binding protein RhoB-like	*LOC106607565*	46,401,032–46,403,034	6	Cytoskeleton
Pleckstrin homology and RhoGEF domain containing G1	*plekhg1*	64,024,693–64,178,709	6	Cytoskeleton

^a^Gene identification (Gene ID) from the NCBI database GenBank assembly accession: GCA_000233375.4.

^b^Location in base pair (bp).

^c^Chromosome (Chr).

**Table 4:** Candidate genes for sea lice resistance in rainbow trout*.*

Protein name	Gene ID1	Location (BP)^b^	Chr^c^	Function
Nuclear factor of activated T-cells, cytoplasmic 1-like	*LOC110518416*	*35,501,191–35,592,262*	3	Immune response
Thymocyte selection-associated family member 2	*themist2*	*33,704,133–33,726,370*	4	Immune response
C–C motif chemokine receptor 10	*ccr10*	*21,961,454–21,965,777*	16	Immune response
T-box 21	*tbx21*	*15,560,804–15,578,932*	16	Immune response
Leucine-rich repeat-containing protein 15-like	*LOC110539182*	*11,715,000–11,717,281*	2	Immune response
Toll-like receptor 13	*LOC110490289*	*48,117,536–48,120,927*	15	Immune response
Adhesion G protein-coupled receptor L3	*LOC110531730*	*14,279,162–14,524,070*	9	Immune response
Inhibin Beta A Chain	*inhba*	*36,734,061–36,745,506*	28	Immune response
Interferon gamma 2	*ifng2*	*153,268–183,367*	Unplaced scaffold	Immune response
Heat shock protein family A (Hsp70) member 8	*hspa8*	*17,691,060–17,715,579*	10	Immune response
Fibroblast growth factor	*fgf13*	*26,660,561–26,715,195*	29	Cytoskeleton
Fibroblast growth factor	*fgf11*	*38,417,625–38,489,806*	10	Cytoskeleton
Alpha-actinin-3	*3a, 3b*	*43,817,039–43,841,681*	10	Cytoskeleton
Dipeptidyl peptidase 3	*ddp3*	*33,026,452–33,044,218*	29	Cytoskeleton
ELMO/CED-12 domain-containing 1	*elmod1*	*16,419,827–16,441,415*	10	Cytoskeleton
Cysteine-rich protein 2-like	*LOC106611283*	*38,639,636–38,675,313*	9	Cytoskeleton
Coagulation factor IX-like	*LOC110534144*	*43,984,179–43,991,091*	10	Cytoskeleton
Lysyl oxidase homolog 1 isoform X2	*LOC110526332*	*59,312,382–59,352,343*	26	Cytoskeleton
Putative ferric-chelate reductase 1	*LOC110490455*	*53,972,784–53,985,982*	15	Metalloprotease
AFG3 Like Matrix AAA Peptidase Subunit 2	*afg3l2*	*35,488,672–35,515,514*	28	Metalloprotease
Afg3-Like Protein 1	*LOC110506600*	*19,946,590–19,959,716*	26	Metalloprotease

^a^Gene identification (Gene ID) from the NCBI database GenBank assembly accession: GCA_002163495.1.

^b^Location in base pair (bp).

^c^Chromosome (Chr).

A recent study on gene expression with *C. rogercresseyi* infestation in susceptible and resistant Atlantic salmon indicated that several components of the immune system (inflammatory response, cytokine production, TNF and NF-kappa B signaling and complement activation) and tissue repair are upregulated during infestation^21^. In salmonids, the main response of the immune system to parasites is mediated by T-Helper 1 and T-Helper 2 cells^25^. Thus, genes related to the immune response, either by promoting leukocyte growth or favoring migration or activation are strong candidate genes. For instance, in Atlantic salmon we found, *T-cell activation Rho GTPase-activating protein* (*tagap*) in Ssa6, which participates in the activation and recruitment of T cells by cytokines^26^, and *tenascin R* (*tnr*) in Ssa3, which is an extracellular matrix protein, present in bone marrow, thymus, spleen and lymph nodes^27^. The latter has been described as having an adhesin function favoring the mobility of lymphocytes and lymphoblasts^27,28^. In rainbow trout, we found candidate genes with similar functions, such as *T-box 21* (*tbx21*), also known as *T-bet* (*T-box* expressed *in T cells*), found in *Omy16*. This gene belongs to the s*ub-Tbr1 family*^29^, and generates type 1 immunity and participates in the maturation and migration of T-helper 1 (Th1) cells, which in turn produce *interferon-gamma* (*IFN-γ*). Studies have described *T-bet* expression in NK cells (natural killer), dendritic cells and T CD8+ cells^30,31^.

*Forkhead box protein N1-like* (*foxn1*) present on *Ssa09* of Atlantic salmon is part of a family of genes widely studied in humans, which are related to various functions including cell growth, lymph node development, and T cell differentiation^32^. It has been proposed that *foxn1* has a role in the activation of fibroblast growth factor receptors^32^.

Meanwhile, in rainbow trout on *Omy21*, *serine/threonine-protein phosphatase 2A 56 kDa* was identified, which is described as having participated in cell growth and signaling^33^. Robledo et al.^21^ recently found that in Atlantic salmon, this protein showed the most significant change in the expression differences between healthy skin and skin where sea lice were attached^21^. In Atlantic salmon, we identified *Tripartite motif-containing protein 45* (*trim45*) on *Ssa25* which belongs to a large family of proteins present in diverse organisms that can function as a ligase and can modify ubiquitin and proteins stimulated by *interferon of 15 kDa* (*isg15*)^34^.

Several metalloproteases were found in genomic regions associated with resistance in both species, but for the interest of this study, we focused on GEM-interacting protein which interacts with *rab27a* or its effector in leukocytes. *Rab* is a large family of GTPases responsible for vesicle cellular transport^35^. Deficiencies of this molecule is correlated with immune deficiencies due to the malfunction of cytotoxic activity of T-lymphocytes, natural killer cells and neutrophils^36^.

Considering the importance of cell growth and movement in response to sea lice infestation, the cytoskeleton may play a considerable role in sea lice resistance as well. For Atlantic salmon, genes related to the cytoskeleton, such as *epidermal growth factor* (*egf*) in *Ssa9*, were identified. This gene is part of a superfamily of receptors with tyrosine kinase activity that have been described in a variety of organs with growth promoter functions, cellular differentiation^38^ and could participate in tissue repair by promoting cell growth^29^. In rainbow trout, fibroblast growth factors (*fgf11, fgf13*) located in *Omy10* and *Omy29* respectively, are involved in angiogenesis and pro-inflammatory responses, and were identified as important genes in sea lice resistance in previous transcriptomic studies by Skugor et al. (2009) and Robledo et al. (2018) in Atlantic salmon^21,39^. In addition, *ELMO/CED-12 domain-containing prot 1 w*as identified in *Omy10* in rainbow trout. This protein participates in phagocytosis of apoptotic cells, and in mammals, it also has a role in cell migration^40^. Other cytoskeleton related candidate genes include: *Procollagen galactosyltransferase 1* present in *Ssa6*, *collagen alpha-1 (XXVIII) chain-like* on *Ssa25* and *pleckstrin homology domain-containing family H member 1-like* on *Ssa9*^41^. The top ten SNPs that explained the highest variance for sea lice resistance are located on *Ssa9* in Atlantic salmon, representing the most important QTL in this species. This QTL is harboring the *breast carcinoma-amplified sequence 3 (bcas3)* gene, which in Atlantic salmon codes for a cell migration factor associated with microtubules that favor cellular mobility^42^. Cell migration is generally induced in response to chemotactic signals, which induces changes in the cytoskeleton and extracellular matrix^43^. We also found, the *tripartite motif-containing protein 16-like* on *Omy15,* which is part of the *trim* superfamily and has functions related to cell differentiation, apoptosis, regulation of transcription and signaling pathways^34^. This gene is similar to *Tripartite motif-containing protein 45* present on *Ssa25*. In this region, we also found a locus that codes for *interferon-γ 2* (*ifng2*), which is a cytokine that participates in type 1 immune responses and that favors the presentation of antigens and activation of macrophages^44^. On this same chromosome (*Omy15*), we also identified *putative ferric-chelate reductase 1 (frrs1),* which functions in the fixation of iron in teleosts ^45^. Robledo et al.^21^ identified *heme*-*binding protein* 2 (*hebp2*) as a gene involved in Atlantic salmon sea lice resistance, which has an iron-binding function. Different authors^46,47^ have stated that decreasing the availability of iron can be part of a nutritional defense mechanism against sea lice infestation.

### Comparative genomics

**Figure 4:**
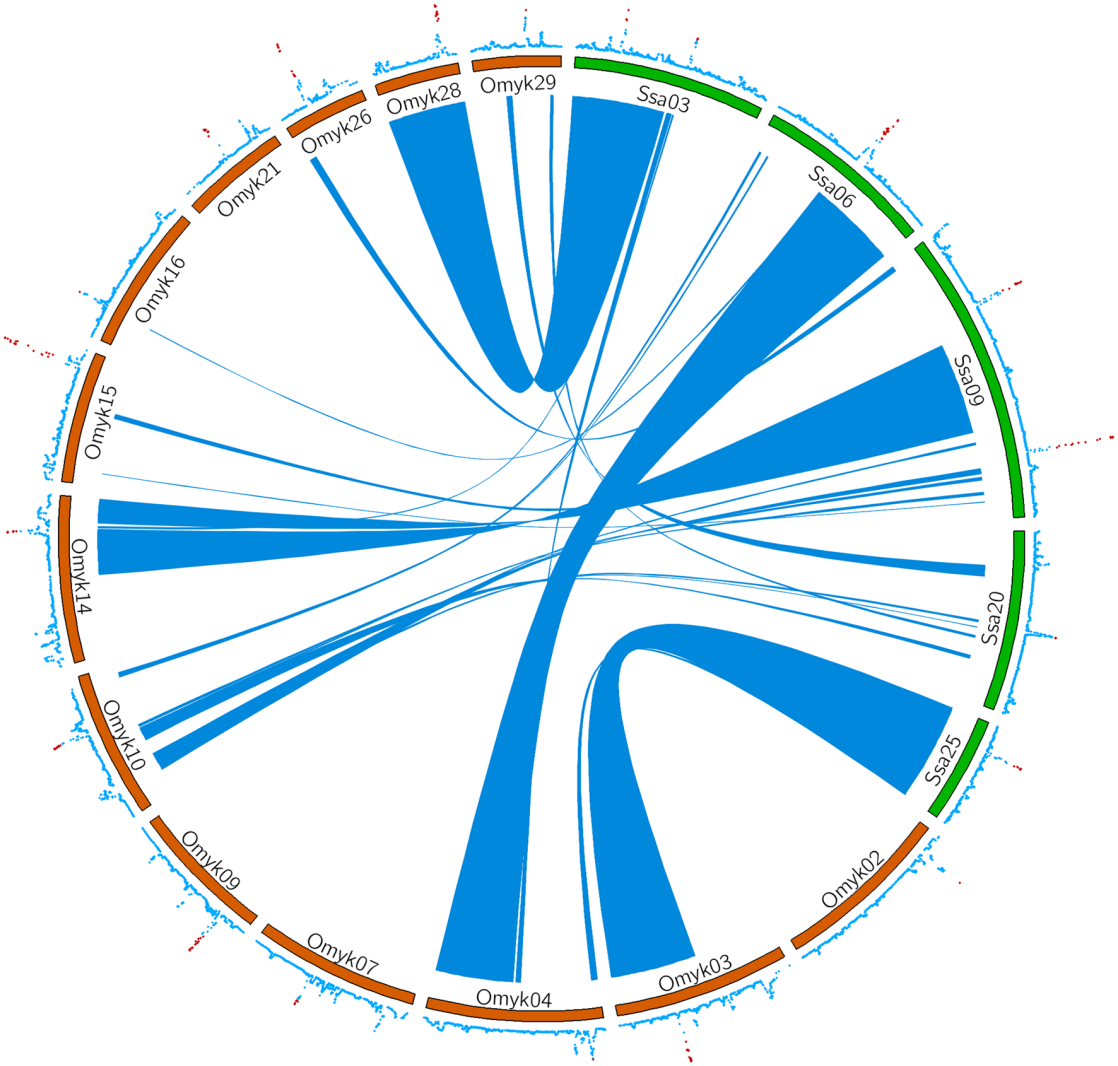
A circos plot for genomic regions explaining more than 1% of the genetic variance for sea lice resistance in Atlantic salmon and rainbow trout. The inner ribbons mark syntenic regions between Atlantic salmon (green and labeled *Ssa*) and rainbow trout (orange and labeled *Omyk*) chromosomes. Genetic variance explained by 20 SNP windows obtained from the wssGWAS analysis are plotted on the outer ring, with the most important windows plotted in red (windows explaining ≥ 1% of the genetic variance).

The comparative genomic analyses performed show regions of synteny between chromosomes associated with sea lice resistance in Atlantic salmon and rainbow trout (Fig. 4). Thus, there are homologous regions which are associated with the trait and share similarities between chromosomes from both species. However, there were no obvious shared regions associated to sea lice resistance within species (i.e. homeologous regions). The examined populations shared homeologous regions harboring genes controlling resistance, which might suggest similar genomic regions involved in the regulation of resistance in the two species. For example, *Ssa3* (Atlantic salmon) shares extensive homology with *Omy28* (rainbow trout) and *Ssa25* with *Omy3*. In addition, when performing the search for orthologue genes (Supplementary Table S2), we found uncharacterized proteins in both species, which shared functionality identified by genomic ontology.

We determined 15 orthogroups that were present in QTLs for sea lice resistance and were shared both within and between species (Supplementary Table S2). These orthogroups were classified according to gene ontology annotations^48^. One of the most interesting groups is orthogroup 12 which contained *lysophosphatidic acid receptor 2-like* (*lpa2*) in Atlantic salmon, and a *G-protein coupled receptor 12-like* in rainbow trout. This orthogroup shares the same GO categories (GO: 0004930, GO: 0007186, GO: 0016021, GO: 0070915, GO: 0007165, GO: 0016020) related to the receptor signaling pathway associated with protein G. The activation of LPA_2_ participates in multiple biological processes, such as cytoskeleton modification via actin fiber formation^49^ and have a role in the activation of *related adhesion focal tyrosine kinase* (*raftk*)^50^, which in turn participates like a stimulating factor for monocytes and macrophages^51^. In orthogroup 13, we identified *dual-specificity protein phosphatase 10-like* (*dusp10*) in Atlantic salmon and *dual-specificity protein phosphatase 8* (*dusp8*) in rainbow trout. These genes might have a similar function in both species, which is most likely related to modulating p38^52^ within the MAPK cascade^53^, a pathway of pro-inflammatory regulators. It has been previously shown that the lice resistant individuals have an up regulated production of pro inflammatory genes than most susceptible fish^54^. The other orthogroups found here did not show a clear relationship with sea lice resistance (Supplementary Table S2).

## Conclusion

The GWAS performed here for Atlantic salmon and rainbow trout made it possible to compare the genetic basis of sea lice resistance in both species. We present novel information about resistance to sea lice in both species. Our results suggest that resistance might be mediated by genes controlling leukocyte response and the cytoskeleton, which promote cell mobility and repair of the wound. The analysis of orthologous proteins provided few characterized proteins. Therefore, further investigations are needed to better annotate genes and generate advances in the elucidation of the genetics behind resistance to *Caligus rogercresseyi* and other important traits in salmonids. We found uncharacterized common genes classified under similar mechanisms by GO terms that could explain resistance in both species. These results suggest that similar mechanisms may regulate sea lice resistance in Atlantic salmon and rainbow trout. Our results provide further knowledge to help establish better control and treatment measures for one of the most important parasitic diseases affecting Atlantic salmon and rainbow trout aquaculture.

## Material and methods

### Experimental animals

All experiments were performed under relevant guidelines and regulations and were approved by the Institutional Committee for Animal Care and Use of the University of Chile (Certificate N 17,041-VET-UCH).

### Sea lice challenge in rainbow trout

The fish for this study belong to a rainbow trout breeding population established in 1998 by Aguas Claras S.A., at Quetroleufu, IX Region, Chile, and currently owned by EFFIGEN S.A. (Puerto Montt, Chile). The population of this study were from the year-class 2011, which has undergone three generations of selection growth, carcass quality and others traits of interest. The details of the population management and breeding program were described by Yoshida et al.^55^. For this study, a total of 2588 PIT (Passive Integrated Transponder) -tagged rainbow trout, originated from 105 maternal full-sib families from the 2012 year-class, were used. For the challenge, the fish were separated into three different tanks so that each family was equally represented in each tank. The *C. rogercresseyi* infestation was conducted with a total of 105,600 copepodites, i.e., an infestation pressure of ~ 40 copepods/fish, which were produced in vitro from ovigerous females. The infestation consisted of depositing the copepodites in each one of the three test tanks, stopping the water flow and keeping the room in darkness for 6 h. On the sixth day after infestation, parasite counting on all fins was performed and caudal fins were sampled for genetic analysis. All fish were euthanized and fins were examined for parasite count using a stereoscopic magnifying glass. Body weight was also recorded for each animal at the end of the challenge.

### Sea lice challenge in Atlantic salmon

A total of 2559 Atlantic salmon smolts belonging to 118 maternal full-sib families from the 2010 year-class of Salmones Chaicas S.A. (Puerto Montt, Chile), were challenged with *C. rogercresseyi*. The fish were PIT-tagged, acclimated and distributed into three tanks as described in previous studies^9,10^. Infestation with the parasite was carried out using 13–24 copepods per fish, stopping the water flow for 6 h after the infestation. The challenge lasted 6 days, then the fish were euthanized and the sea lice were counted on all of the fins. A sample of the caudal fin was taken for genetic analysis and the body weight of each fish was measured at the end of the challenge.

### Genotyping

Genomic DNA was extracted from the caudal fin of each challenged fish using the DNeasy Blood & Kit tissue kit (Qiagen), following the manufacturer's instructions. The 2628 Atlantic salmon samples were genotyped using a custom Affymetrix^®^ 50 K Axiom^®^ myDesign™ Genotyping Array designed by AquaInnovo and the University of Chile^56^, while the 2643 rainbow trout samples were genotyped with a 57 K SNP array developed by the United State Department of Agriculture (USDA)^57^. Quality control of the genotypes was carried out in PLINK v1.90b3.34. SNPs with a call rate ≤ 0.95, a Minor Allele Frequency (MAF) < 0.05 and those that were not in Hardy–Weinberg equilibrium (p < $1\times 10^{-6}$) were discarded. Individuals were filtered if they had a call rate ≤ 0.95. All the SNPs and fish that passed quality control, were used for downstream analysis.

### Genomic association analysis

Resistance to *C. rogercresseyi* was defined as follows, according to Ødegård et al.^11^:

$$LogLD={log}_{e}\left(\frac{LC+1}{\sqrt[3]{} {BW}^{2}}\right)$$

where LD is the *C. rogercresseyi* density defined as the lice count (LC) on each fish at the end of the experimental challenge plus a unity, divided by the cube root of the squared body weight of the fish on the same day (BW), which is an approximation of the surface of the skin of each fish. The logarithm of LD was used as it has an approximately normal distribution.

A weighted single-step genomic association study (wssGWAS)^58^ was used to identify associations between SNPs and resistance to *C. rogercresseyi* in both species, using the BLUPF90 family of programs^59^. This approach uses a combination of both genomic and pedigree matrixes. Genotype and pedigree information were used to generate the **H** kinship matrix^60^, as defined in the following equation:

$${H}^{-1}={A}^{-1}+\left[\begin{array}{cc}0& 0\\ 0& {G}^{-1}-{A}_{22}^{-1}\end{array}\right]$$

where ${A}^{-1}$ is the inverse of the relationship matrix for all the animals, constructed from the pedigree, ${A}_{22}^{-1}$ is the inverse of the pedigree relationship matrix for the genotyped animals, and ${G}^{-1}$ is the inverse of the genomic relationship matrix for the genotyped animals. The SNPs were weighted with equal value and assigned the constant 1 to perform the single-step GWAS method. For the weighted single-step GWAS method, the markers were assigned to weights estimated by the previous method. The association analysis for both species were performed using the following mixed linear model y = ***X***b + **Z**a + e, where **y** is the vector of phenotypic values (LogLD); **b** is the vector of fixed effects (tank); **a** is the vector of random animal effects, considering the structure of covariance between individuals established by matrix **H**, and **e** is the vector of random residuals; **X** and **Z** are the incidence matrices for fixed and random animal effects, respectively.

To identify the regions of the genome associated with resistance, we generated windows of 20 adjacent SNPs. Thereafter, if a window explained more than 1% of the genetic variance, it was considered associated with the trait. We also estimated the p-values for individual SNP-trait associations by using BLUPF90 software^61^.

### Genome comparison

The rainbow trout (GCF_002163495.1)^62^ and Atlantic salmon (GCF_000233375.1)^63^ genomes were downloaded from the NCBI database and the subset for chromosomes associated with sea lice resistance was aligned with software Samtools ^64^. Synteny between the chromosomes was identified by aligning sequences using the program Symap v3.4^65^. Circos^66^ was used to visualize the relationships between genomic regions associated to sea lice resistance in rainbow trout and Atlantic salmon chromosomes.

### Candidate genes

The 71 pb flanking sequences surrounding SNPs associated with sea lice resistance were aligned to the most recent reference genomes of Atlantic salmon and rainbow trout using BLASTn^67^. Sequences covering 1 Mb, flanking the associated SNPs (0.5 Mb downstream and 0.5 Mb upstream), were saved in the FASTA format. BLASTx was then used to identify coding sequences for proteins in these 1 Mb associated windows. Blast2GO^68^ was used in parallel with the FASTA file to identify proteins and classify them by function. For both species, the reference genome of *Danio rerio* (GenBank Assembly Accession: GCA_000002035.4) was used to annotate proteins that were not characterized in the rainbow trout or Atlantic salmon reference genomes. To identify orthologous proteins/genes between species, the OrthoFinder^69^ program was used with the FASTA sequences obtained with BLASTx.

### Ethics approval and consent to participate

All the experimental challenges were approved by the Institutional Committee for Animal Care and Use of the University of Chile (Certificate N 17,041-VET-UCH). We also confirm that the study was carried out in compliance with the ARRIVE guidelines.

## Supplementary Information

Supplementary Information.

## Data Availability

Atlantic salmon and rainbow trout phenotype and genotype data are available at https://figshare.com/articles/Compative_genomic_of_O_mykiss_and_S_salar_for_resistance_to_Sea_lice/7676147 and https://figshare.com/projects/Compative_genomic_of_O_mykiss_and_S_salar_for_resistace_to_Sea_lice/70904, respectively.
