# Supplementary material for: Genome-scale comparative analysis for host resistance against sea lice between Atlantic salmon and rainbow trout
Source: Sci Rep. 2021 Jun 24;11:13231. doi: 10.1038/s41598-021-92425-3 (PMC8225872; doi:10.1038/s41598-021-92425-3)

**Genome-scale comparative analysis for host resistance against sea lice between Atlantic salmon and rainbow trout**

Pablo Cáceres, Agustín Barría, Kris A. Christensen, Liane N. Bassini, Katharina Correa, Baltasar García, Jean P. Lhorente, José M. Yáñez

**Supplementary Table 1**. Window position and genetic variance of representative SNP.

| Window Position (bp)^1^ | Chr^2^ | First and last SNP of window | Var (%)^3^ |
| --- | --- | --- | --- |
|  |  | **Atlantic Salmon** |  |
| 111398941-112068765 | 9 | Affx-93376378-Affx-93407247 | 3.00 |
| 46812870-48951308 | 6 | Affx-93402585-Affx-93349216 | 1.97 |
| 38862311-39791664 | 9 | Affx-93301094-Affx-93369286 | 1.76 |
| 21387020-22266613 | 3 | Affx-93351937-Affx-93441285 | 1.48 |
| 13302321-13992142 | 25 | Affx-93449253-Affx-93379811 | 1.33 |
| 53395987-54096750 | 3 | Affx-93397375-Affx-93327854 | 1.08 |
| 47471286-48390124 | 20 | Affx-93269128-Affx-93365186 | 1.05 |
|  |  | **Rainbow trout** |  |
| 55155679-55750071 | 15 | Affx-88957834-Affx-88937607 | 2.77 |
| 14742278- 15862261 | 26 | Affx-88926342-Affx-88913419 | 2.35 |
| 35337013- 36760480 | 28 | Affx-88961757-Affx-88909402 | 1.77 |
| 57059237- 57975684 | 3 | Affx-88911532-Affx-88917594 | 1.73 |
| 14313209- 15091943 | 9 | Affx-88944322-Affx-88927798 | 1.67 |
| 10428810- 11808090 | 2 | Affx-88922063-Affx-88937734 | 1.66 |
| 62615440- 63610148 | 14 | Affx-88942436-Affx-88960365 | 1.43 |
| 27521395-28664751 | 21 | Affx-88932888-Affx-88952962 | 1.31 |
| 43708499-44788897 | 10 | Affx-88912735-Affx-88908041 | 1.29 |
| 26370287-27397531 | 29 | Affx-88925775-Affx-88920967 | 1.25 |
| 50072755-50809742 | 7 | Affx-88956194-Affx-88906442 | 1.17 |
| 15571716-16056405 | 16 | Affx-88940047-Affx-88947010 | 1.07 |
| 7055909-8035171 | 4 | Affx-88935786-Affx-88957720 | 1.03 |

^1^Window position in base pair (BP).

^2^Chromosome (Chr).

^3^ Variance (Var)

**Supplementary Table 2.** Genes present in main orthogroups.

| OG^1^ | Species | Gene | Description | GO term | Chr^2^ |
| --- | --- | --- | --- | --- | --- |
| 01 | *S. salar*  *O. mykiss* | *ZSCAN29, zg57, ZKSCAN8*  *ZIC3* | zinc finger and SCAN domain  zinc finger protein | [GO:0003676](https://www.ebi.ac.uk/QuickGO/term/GO:0003676)  [GO:0003676](https://www.ebi.ac.uk/QuickGO/term/GO:0003676)  [GO:0010469](https://www.ebi.ac.uk/QuickGO/term/GO:0010469)  [GO:0008083](https://www.ebi.ac.uk/QuickGO/term/GO:0008083) | 3,20,3,6  10 |
| 02 | *S. salar*  *O. mykiss* | LOC106599059  GSONMT000685230012^3^ | piggyBac transposable element-derived protein 4-like  uncharacterized | [GO:0003677](https://www.ebi.ac.uk/QuickGO/term/GO:0003677)  [GO:0006313](https://www.ebi.ac.uk/QuickGO/term/GO:0006313)  [GO:0015074](https://www.ebi.ac.uk/QuickGO/term/GO:0015074)  [GO:0003677](https://www.ebi.ac.uk/QuickGO/term/GO:0003677)  [GO:0003779](https://www.ebi.ac.uk/QuickGO/term/GO:0003779)  [GO:0006313](https://www.ebi.ac.uk/QuickGO/term/GO:0006313)  [GO:0015074](https://www.ebi.ac.uk/QuickGO/term/GO:0015074)  [GO:0016192](https://www.ebi.ac.uk/QuickGO/term/GO:0016192)  [GO:0045010](https://www.ebi.ac.uk/QuickGO/term/GO:0045010) | 3  UN^3^ |
| 03 | *S. salar*  *O. mykiss* | *TCB1*  CDQ96296 | Transposable element Tcb1 transposase  unnamed protein product | [GO:0003677](https://www.ebi.ac.uk/QuickGO/term/GO:0003677)  [GO:0006313](https://www.ebi.ac.uk/QuickGO/term/GO:0006313)  [GO:0015074](https://www.ebi.ac.uk/QuickGO/term/GO:0015074)  [GO:0003677](https://www.ebi.ac.uk/QuickGO/term/GO:0003677)  [GO:0006313](https://www.ebi.ac.uk/QuickGO/term/GO:0006313)  [GO:0015074](https://www.ebi.ac.uk/QuickGO/term/GO:0015074) | UN  UN |
| 04 | *S. salar*  *O. mykiss* | AKP40998  FR943085.1 | ORF2 protein  uncharacterized | Reverse transcriptase  Reverse transcriptase | UN  UN |
| 05 | *S. salar*  *O. mykiss* | LOC106566723 LOC106571716 LOC106584292 LOC106569845  LOC110485914 | uncharacterized protein K02A2.6-like  uncharacterized protein K02A2.6-like | [GO:0003676](https://www.ebi.ac.uk/QuickGO/term/GO:0003676)  [GO:0015074](https://www.ebi.ac.uk/QuickGO/term/GO:0015074) | 13,15,23,  14  23  13  13 |
| 06 | *S. salar*  *O. mykiss* | ABV31711.1  CDQ65039 | Transposase  Transposase | [GO:0003677](https://www.ebi.ac.uk/QuickGO/term/GO:0003677)  [GO:0006313](https://www.ebi.ac.uk/QuickGO/term/GO:0006313)  [GO:0015074](https://www.ebi.ac.uk/QuickGO/term/GO:0015074)  [GO:0003677](https://www.ebi.ac.uk/QuickGO/term/GO:0003677)  [GO:0006313](https://www.ebi.ac.uk/QuickGO/term/GO:0006313)  [GO:0015074](https://www.ebi.ac.uk/QuickGO/term/GO:0015074) | UN  UN |
| 07 | *S. salar*  *O. mykiss* | LOC106600457  *atp8a1* | uncharacterized protein  Phospholipid-transporting ATPase | [GO:0045332](https://www.ebi.ac.uk/QuickGO/term/GO:0045332)  [GO:0000166](https://www.ebi.ac.uk/QuickGO/term/GO:0000166)  [GO:0000287](https://www.ebi.ac.uk/QuickGO/term/GO:0000287)  [GO:0004012](https://www.ebi.ac.uk/QuickGO/term/GO:0004012)  [GO:0005524](https://www.ebi.ac.uk/QuickGO/term/GO:0005524)  [GO:0015914](https://www.ebi.ac.uk/QuickGO/term/GO:0015914)  [GO:0016021](https://www.ebi.ac.uk/QuickGO/term/GO:0016021)  [GO:0016020](https://www.ebi.ac.uk/QuickGO/term/GO:0016020) | 3  14 |
| 08 | *S. salar*  *O. mykiss* | LOC100194703  FR975430.1 | Transposase  uncharacterized | [GO:0003677](https://www.ebi.ac.uk/QuickGO/term/GO:0003677)  [GO:0006313](https://www.ebi.ac.uk/QuickGO/term/GO:0006313)  [GO:0015074](https://www.ebi.ac.uk/QuickGO/term/GO:0015074) | 13  UN |
| 09 | *S. salar*  *O. mykiss* | LOC100380853  CDQ96836.1 | Transposon Tf2-1 polyprotein  uncharacterized | [GO:0003676](https://www.ebi.ac.uk/QuickGO/term/GO:0003676)  [GO:0015074](https://www.ebi.ac.uk/QuickGO/term/GO:0015074)  [GO:0016020](https://www.ebi.ac.uk/QuickGO/term/GO:0016020)  [GO:0016021](https://www.ebi.ac.uk/QuickGO/term/GO:0016021)  [GO:0003676](https://www.ebi.ac.uk/QuickGO/term/GO:0003676)  [GO:0015074](https://www.ebi.ac.uk/QuickGO/term/GO:0015074) | 9  UN |
| 10 | *S. salar*  *O. mykiss* | LOC106582599  FR904579.1 | uncharacterized  uncharacterized | [GO:0003676](https://www.ebi.ac.uk/QuickGO/term/GO:0003676)  [GO:0008270](https://www.ebi.ac.uk/QuickGO/term/GO:0008270) | 22  UN |
| 11 | *S. salar*  *O. mykiss* | LOC106612089  LOC106568774  LOC106566110  LOC110493928  LOC110497478 | uncharacterized  uncharacterized  uncharacterized  uncharacterized  uncharacterized | [GO:0003676](https://www.ebi.ac.uk/QuickGO/term/GO:0003676)  [GO:0015074](https://www.ebi.ac.uk/QuickGO/term/GO:0015074) | 1,14,1  14  1  17,19  19 |
| 12 | *S. salar*  *O. mykiss* | LOC106600920  LOC110510161  FR905264.1 | lysophosphatidic acid receptor 2-like  G-protein coupled receptor 12-like  uncharacterized | [GO:0004930](https://www.ebi.ac.uk/QuickGO/term/GO:0004930)  [GO:0007186](https://www.ebi.ac.uk/QuickGO/term/GO:0007186)  [GO:0016021](https://www.ebi.ac.uk/QuickGO/term/GO:0016021)  [GO:0070915](https://www.ebi.ac.uk/QuickGO/term/GO:0070915)  [GO:0007165](https://www.ebi.ac.uk/QuickGO/term/GO:0007165)  [GO:0016020](https://www.ebi.ac.uk/QuickGO/term/GO:0016020)  [GO:0004930](https://www.ebi.ac.uk/QuickGO/term/GO:0004930)  [GO:0007186](https://www.ebi.ac.uk/QuickGO/term/GO:0007186)  [GO:0016021](https://www.ebi.ac.uk/QuickGO/term/GO:0016021)  [GO:0016020](https://www.ebi.ac.uk/QuickGO/term/GO:0016020)  [GO:0007165](https://www.ebi.ac.uk/QuickGO/term/GO:0007165) | 3,29  29  UN |
| 13 | *S. salar*  *O. mykiss* | dusp10  LOC110521303  dusp8 | dual specificity protein phosphatase 10-like  dual specificity protein phosphatase 8/ like | [GO:0000188](https://www.ebi.ac.uk/QuickGO/term/GO:0000188)  [GO:0035335](https://www.ebi.ac.uk/QuickGO/term/GO:0035335)  [GO:0004725](https://www.ebi.ac.uk/QuickGO/term/GO:0004725)  [GO:0006470](https://www.ebi.ac.uk/QuickGO/term/GO:0006470)  [GO:0008138](https://www.ebi.ac.uk/QuickGO/term/GO:0008138)  [GO:0016311](https://www.ebi.ac.uk/QuickGO/term/GO:0016311)  [GO:0016791](https://www.ebi.ac.uk/QuickGO/term/GO:0016791)  [GO:0017017](https://www.ebi.ac.uk/QuickGO/term/GO:0017017)  [GO:0016787](https://www.ebi.ac.uk/QuickGO/term/GO:0016787) | 6  4  2 |
| 14 | *S. salar*  *O. mykiss* | LOC106585798  LOC110514228 | uncharacterized  uncharacterized | [GO:0003676](https://www.ebi.ac.uk/QuickGO/term/GO:0003676)  [GO:0003700](https://www.ebi.ac.uk/QuickGO/term/GO:0003700)  [GO:0006355](https://www.ebi.ac.uk/QuickGO/term/GO:0006355)  [GO:0008270](https://www.ebi.ac.uk/QuickGO/term/GO:0008270)  [GO:0015074](https://www.ebi.ac.uk/QuickGO/term/GO:0015074) | 24  UN |
| 15 | *S. salar*  *O. mykiss* | LOC106578697  AGL94530.1 | general transcription factor II-I repeat domain-containing protein 2-like  putative general transcription factor II-I repeat domain containing protein 2-like protein |  | 19  Y |

^1^Orthogroup.

^2^Chromosome.

^3^ Unplaced Scaffold

^4^ Gene name in uniprot database.

**Supplementary Figure 1**. Weighted single-step GWAS (wssGWAS) results for Log sea lice density (LogLD) in Atlantic salmon. The Manhattan plot represents the –log_10_(p-value) per SNP, the blue and red horizontal lines indicate chromosome- and genome-wide significance threshold, respectively.


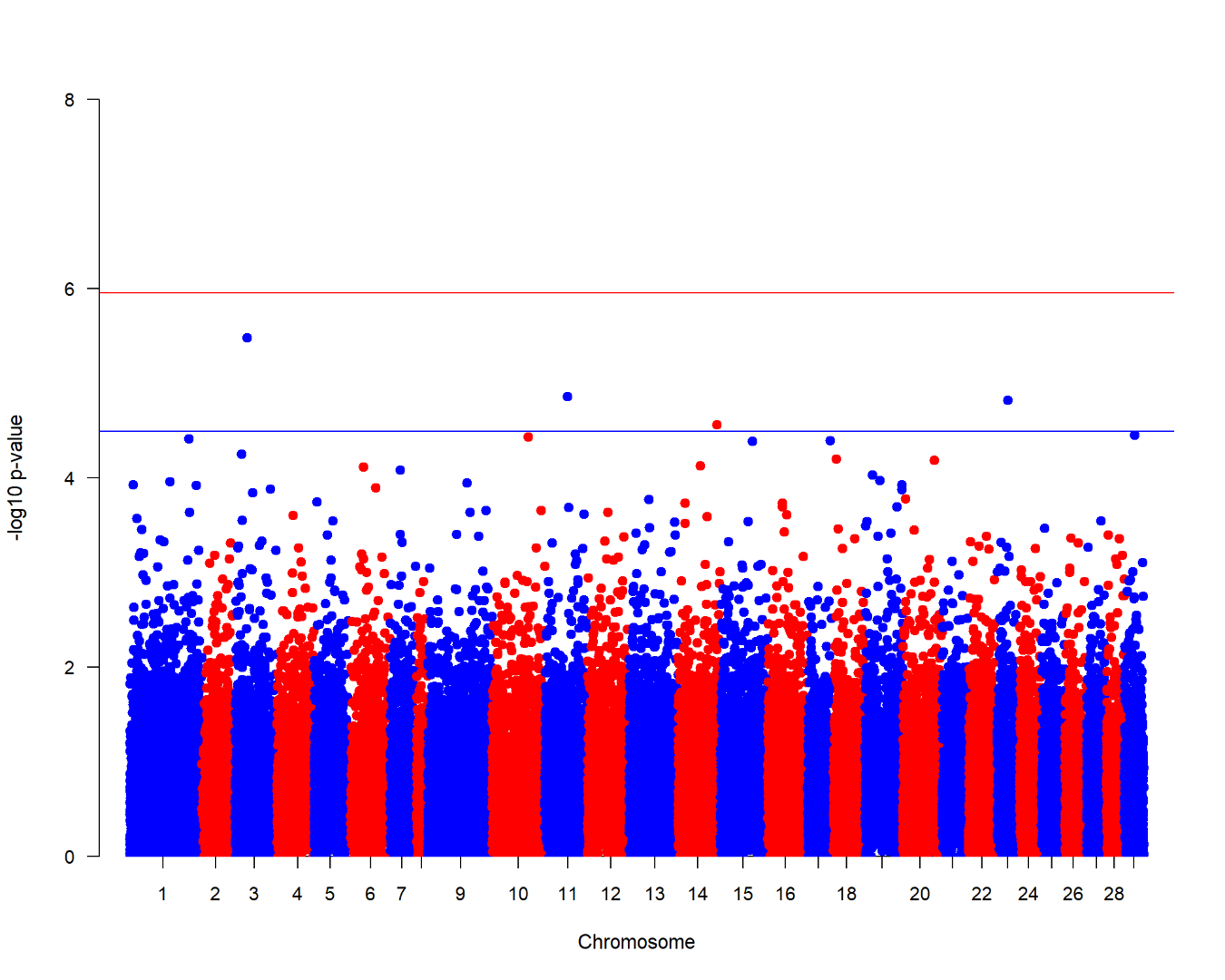


**Supplementary Figure 2**. Weighted single-step GWAS (wssGWAS) results for Log sea lice density (LogLD) in rainbow trout. The Manhattan plot represents the –log_10_(p-value) per SNP, the blue and red horizontal lines indicate chromosome- and genome-wide significance threshold, respectively.


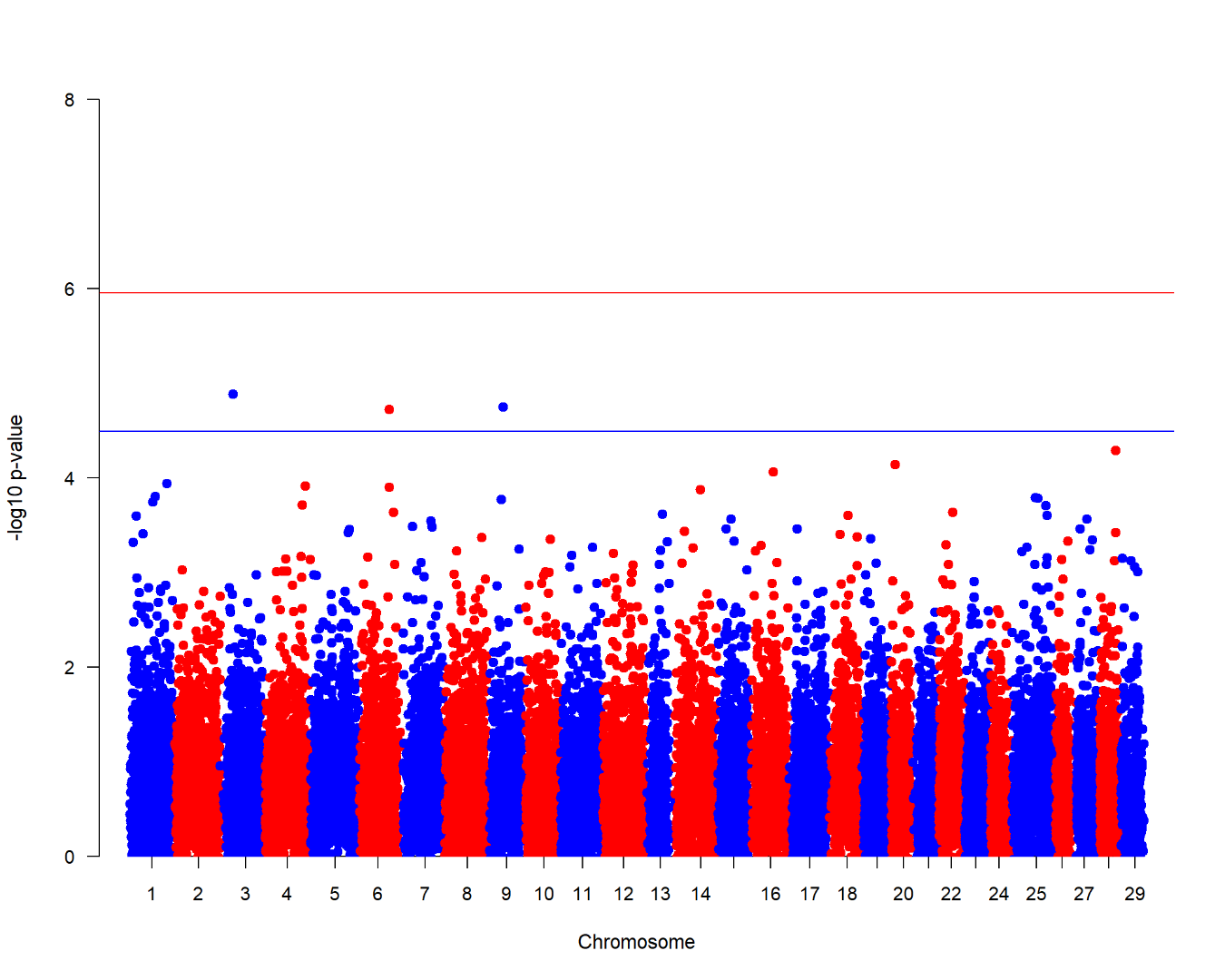

Supplement: Supplementary file 1 — Supplementary Information. [file 41598_2021_92425_MOESM1_ESM.docx]
